# Clinicopathological differences of high *Fusobacterium nucleatum* levels in colorectal cancer: A review and meta-analysis

**DOI:** 10.3389/fmicb.2022.945463

**Published:** 2022-11-04

**Authors:** Yi Wang, Yuting Wen, Jiayin Wang, Xin Lai, Ying Xu, Xuanping Zhang, Xiaoyan Zhu, Chenglin Ruan, Yao Huang

**Affiliations:** ^1^School of Computer Science and Technology, Xi’an Jiaotong University, Xi’an, Shaanxi, China; ^2^Department of Pathology, Xi’an Ninth Hospital Affiliated to Medical College of Xi’an Jiaotong University, Xi’an, Shaanxi, China

**Keywords:** cancer, colorectal cancer, meta-analysis, *Fusobacterium nucleatum*, review

## Abstract

**Objective:**

To systematically evaluate the significance of *Fusobacterium nucleatum* (Fn) levels the clinicopathological impacts of cancer.

**Methods:**

Literature from Pubmed, Embase, and Web of Science was retrieved to collect all English literatures on the correlation between Fn and cancer, and the quality of literatures collected was assessed based on the Newcastle-Ottawa Quality Assessment Scale. The heterogeneity and sensitivity were detected by Stata 14.0 software, and the correlation between Fn and cancer clinicopathological as the effect variables was assessed according to the odds ratio (OR) and 95% confidence interval (CI). The forest plot was drawn.

**Results:**

A total of 19 articles meeting the inclusion criteria were selected. The incidence of Fn prevalence varied considerably (range: 6.1 to 83.3%) and was greater than 10% in 13 of 19 studies. Compared with those with no/low Fn levels, the high levels of Fn was positively associated with vascular invasion, nerve invasion, depth of invasion, and distant metastasis [vascular invasion: OR = 1.66, 95%CI(1.07, 2.57), *I*^2^ = 21.9%, fixed effect model; nerve invasion: OR = 1.36, 95%CI(1.00, 1.84), *I*^2^ = 43.1%, fixed effect model; infiltration depth: OR = 1.94, 95%CI(1.20, 3.15), *I*^2^ = 67.2%, random effect model; distant metastasis: OR = 1.80, 95%CI(1.23, 2.64), *I*^2^ = 3.4%, fixed effect model]. Patients with MLH1 methylation always present a higher Fn levels than those without methylation [OR = 2.53, 95%CI(1.42, 4.53), *P* = 0.01, *I*^2^ = 57.5%, random effect model]. Further, Fn was associatedwith the molecular characteristics of cancers [MSI-H Vs. MSS/MSI-low: OR = 2.92, 95%CI(1.61, 5.32), *P* = 0.01, *I*^2^ = 63.2%, random effect model; High Vs. Low/Negative CIMP: OR = 2.23, 95%CI(1.64, 3.03), *P* = 0.01, *I*^2^ = 64.2%, random effect model; KRAS mutation Vs. wild-type: OR = 1.24, 95%CI(1.04, 1.48), *P* = 0.02, *I*^2^ = 27.0%, fixed effect model; Present Vs. Abscent BRAF mutations: OR = 1.88, 95%CI(1.44, 2.45), *P* = 0.01, *I*^2^ = 24.2%, fixed effect model]. The cancer patients with high levels of Fn often have worse RFS than those with no/low Fn levels[OR = 1.14, 95%CI(0.61, 1.68), *P* = 0.01, *I*^2^ = 80.7%, random effect model].

**Conclusion:**

This review and meta-analysis showed that Fn could be used to predict unfavorable prognosis and function as potential prognostic biomarkers in colorectal cancer (CRC). Our data may have implications for targeting Fn to develop strategies for cancer prevention and treatment.

## Introduction

Colorectal cancer (CRC) is the third most common cancer and the fourth most common cause of global cancer deaths (Bray et al., 2018). Although great improvements in the diagnosis and treatment of CRC have been made, the prognosis is still not promising (Zhang et al., 2021). The prognosis of CRC depends on the clinicopathologic stage at the time of diagnosis. However, the disease stage alone cannot accurately predict the prognosis of individual patients (Chao et al., 2020). Therefore, identifying potential risk factors related to CRC prognosis is important in improving the survival of CRC patients.

Recent studies have shown a genera-specific shift in abundance between healthy microbiota present in normal tissues and potentially cancer-related bacteria in CRC (de Carvalho et al., 2019). Compared to normal tissues, Fusobacteria were enriched in human cancer tissues, especially *Fusobacterium nucleatum* (Fn; de Carvalho et al., 2019). Fn is a Gram-negative anaerobic microorganism that is indigenous to the human oral cavity (Kostic et al., 2012). The existing studies have reported that Fn may cause CRC by inducing inflammation and suppressing host immunity, possibly through modulating the E-cadherin/β-catenin pathway via FadA adhesion in Fn (Rubinstein et al., 2013). A previous work showed that the levels of Fn associates with CRC progression and it could be used as a prognosis biomarker for cancer (Gethings-Behncke et al., 2020). However, the correlation between the high levels of Fn and the clinicopathology and prognosis characteristics of CRC is still controversial.

Consider the case of clinicopathology, for example. A previous cohort study (Chen et al., 2020) showed that high Fn levels were positively associated with vascular and nerve invasion of cancer tissues, while another study (Lee et al., 2021) showed that Fn levels were not related to them. A study of Mima et al. (2016) showed the level of Fn in CRC tissue was associated with shorter survival, and may potentially serve as a prognostic biomarker. Contrarily, another study reported tumors with high levels of Fn had a better prognosis than those with low or negative levels of Fn in non-sigmoid colon cancers (Jeong et al., 2019). Yamamoto et al. (2021) confirmed that KRAS and BRAF mutation were related to high Fn levels, while Ito et al. (2015) believed that they were unrelated.

Based on the above controversial statements, we conducted a large sample integration study in this study to clarify the role of Fn infection in colorectal cancer patients.

## Materials and methods

### Search strategy and study identification

A literature search on PubMed, Embase, and Web of Science was conducted to identify all primary studies relating to Fn and patient clinicopathological and prognosis published before 2022. The literature search used broad search terms that could ensure the inclusion of relevant literatures. We also reviewed the reference lists of the articles identified in the primary search for additional relevant studies. A specific search strategy was devised to include at least one keyword or Medical Subject Heading (MeSH) term from each of the following: (i) Neoplasia or Neoplasias or Neoplasm or Tumors or Cancer or Cancers or Malignancy or Malignancies or Malignant Neoplasms or Malignant Neoplasm or Neoplasm, Malignant or Neoplasms, Malignant or Benign Neoplasms or Neoplasms, Benign or Benign Neoplasm or Neoplasm, Benign; (ii) Bacteria or Fusobacteriaor or Fusobacterium or *Fusobacterium nucleatum*.

### Inclusion/exclusion criteria

The inclusion criteria were as follows: (1) Tissue specimens from humans were obtained after surgical resection for the primary tumors. None of the patients received radiochemotherapy before surgery. (2) Original research articles were included in the final analysis. (3) Experiments were conducted using conventional quantitative polymerase chain reaction (qPCR) or 16S rRNA sequencing methodology.

The exclusion criteria are as follows: (1) Letters, abstracts, books, short conference abstracts/proceedings, and posters. (2) Cell and animal studies. (3) The publications on a small-sized corhort on the same topic from the same team (shared overlapped participants with large-sized studies). (4) Low quality studies (score < 5). (5) Neoadjuvant or adjuvant chemoradiotherapy.

### Data extraction

The study design information and outcome data were independently extracted by two investigators (Yi Wang and Yuting Wen). We extracted data on the following: study characteristics (e.g., study design, year of publication, number of samples, types of cancer, population, clinicopathological features of patients, survival analysis), measurement methods, outcome measures, and statistical analysis. We assessed study quality using items from the Newcastle-Ottawa Quality Assessment Scale (NOS; Stang, 2010; Supplementary Table S1).

### Statistical analysis

All statistical analyses were performed using Stata 14.0 (Stata Corp., College Station, TX, United States). Data were extracted from the original research articles and converted into 2 × 2 tables. The definitions of study characteristics, measurement methods, outcome measures were set by the authors of each paper, and the data were combined for analysis only if these definitions were sufficiently similar (determined by consensus). Pooled estimates and corresponding 95% CIs were represented by forest plots. Dichotomous variables [Mucus, Signet-ring cells, Lymphatic metastasis, Vascular invasion, Nerve invasion, Peridumoral lymphocytes (present VS. absent); differentiation (medium-low VS. high); infiltration depth (pT3/4 VS. pT1/2); regional lymph node metastasis (pN1/2 VS. pN0); distant metastasis (pM1 VS. pM0); Tumor Node Metastasis (III-IV VS. I-II); MSI (high VS. low); MLH1 methylation, CIMP status (high VS. low/negative); KRAS mutation, BRAF mutation (mutant VS. wild)] were analyzed with odds ratios (ORs) with mean differences at a 95% confidence interval (CI). Continuous variables were analyzed by the HR, and 95% CI was recorded. Recurrence free survival (RFS) was defined as the time from complete remission to recurrence or follow-up deadline.

Statistical heterogeneity across studies was evaluated with the Q statistics (p < 0.05 was considered significant) and the Inconsistency statistics (*I*^2^) (Higgins and Thompson, 2002). *I*^2^ = 0–25%, no heterogeneity; *I*^2^ = 25–50%, moderate heterogeneity; *I*^2^ = 50–75%, large heterogeneity; and *I*^2^ = 75–100%, extreme heterogeneity. If no significant heterogeneity existed, the pooled estimates and the 95% CI were calculated using a fixed-effect model. If a significant heterogeneity was presented (*I*^2^ statistics no less than 50%), then a random-effect model was adopted. Sensitivity analysis was performed to explore the influence of an individual study on the pooled results, by deleting a single study each iteration from the pooled analysis. We used subgroup analysis to evaluate for potential sources of heterogeneity. All important results (p < 0.05) were analyzed using Egger’s and Begg’s tests for publication bias. A p value <0.10 was considered as suggesting the publication bias.

## Results

### Study characteristics

**Figure 1:**
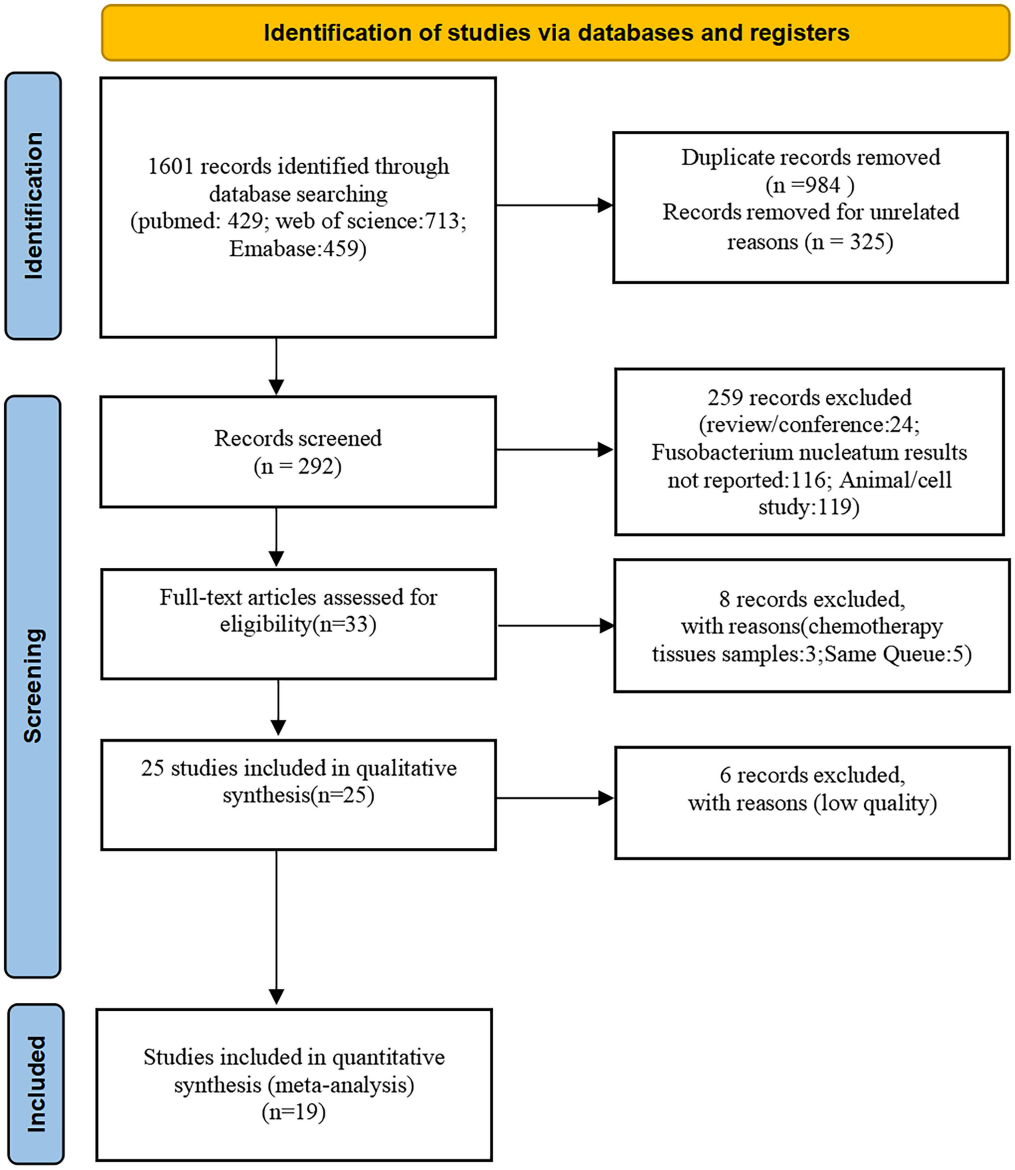
Study selection. A total of 19 eligible studies were included from 1,601 potentially relevant studies identified and reviewed.

The literature screening process was presented in Figure 1 (The PRISMA 2020 statement. doi: 10.1136/BMJ.n71). Initial literature searches retrieved 1,601 articles, from which 984 were screened after excluding duplicates. The 325 citations were removed due to irrelevant publishing types or studies by a screening of the titles and abstracts. A total of 25 full-text articles were subsequently assessed for eligibility and 6 were removed due to low-quality scores (NOS quality scores<3). Finally, a total of 19 studies (Higgins and Thompson, 2002; Tahara et al., 2014; Mitsuhashi et al., 2015; Sun et al., 2016; Park et al., 2017; Yan et al., 2017; Yu et al., 2017; Yamaoka et al., 2018; Chen et al., 2019; Jeong et al., 2019; Kunzmann et al., 2019; Zhang et al., 2019; Boehm et al., 2020; Haruki et al., 2020; Serna et al., 2020; Egger et al., 2021; Lee et al., 2021; Nie et al., 2021; Yamamoto et al., 2021) were included in this meta-analysis which involved 4,920 participants (1,267 Fn-high and 3,653 Fn-no/low, respectively). Basic characteristics and Fn performance in cancer detection of these 19 studies (16 studies on CRC, 2 studies on GC, and 1 study on PAAD) were shown in Table 1. There was no disagreement among the authors as to whether the above studies should be included in this meta-analysis.

**Table 1:** Characteristics of the studies included in the meta-analysis.

					Num. patient (Fn)	
Study	Year	Country	Collection time	Cancer	High	No/Low	Method	TNM	Score
Jeong et al. (2019)	2019	Korea	2005–2012	CRC	204	389	qPCR	II–III	6
Yu et al. (2017)	2017	China	2012–2015	CRC	/	/	16SrRNA	/	9
Park et al. (2017)	2017	Korean	/	CRC	15	145	16SrRNA	I–IV	6
Yan et al. (2017)	2017	China	2007–2015	CRC	187	93	qRT-PCR	III–IV	6
Zhang et al. (2019)	2019	China	2007–2017	CRC	21	73	qPCR	II–III	7
Lee et al. (2021)	2021	Korean	2014–2018	CRC	57	69	qPCR	I–IV	6
Nie et al. (2021)	2021	China	2012–2014	GC	30	31	16SrRNA	I–IV	7
Chao et al. (2020)	2019	China	2017–2019	CRC	25	66	qPCR	II–III	6
Kunzmann et al. (2019)	2019	Czech	2008–2012	CRC	61	129	qPCR	I–IV	7
Mitsuhashi et al. (2015)	2015	Japan	2003–2013	PAAD	25	258	qPCR	I–IV	9
Boehm et al., 2020	2020	Lithuania	/	GC	63	18	qPCR	I–IV	9
Yamaoka et al. (2018)	2018	Japan	1999–2008	CRC	75	15	ddPCR	I–IV	9
Yamamoto et al. (2021)	2021	Japan	2017–2018	CRC	44	156	qPCR	I–IV	9
Serna et al. (2020)	2020	Britain	2007–2018	CRC	73	53	qPCR	/	9
Mima et al. (2016)	2015	United States	~2008	CRC	67	1,002	qPCR	I–IV	7
Tahara et al. (2014)	2014	United States	/	CRC	10	79	qPCR	/	9
Ito et al. (2015)	2015	Japan	/	CRC	143	368	qRT-PCR	I–IV	9
Sun et al. (2016)	2016	China	2009–2010	CRC	118	34	qPCR	I–IV	9
Haruki et al. (2020)	2020	United States	1976–2008	CRC	49	675	qPCR	I–IV	9

CRC, Colorectal cancer; GC, gastric carcinoma; PAAD, pancreatic adenocarcinoma; Fn, Fusobacterium nucleatum; qPCR:Quantitative polymerase chain reaction; qRT-PCR, Quantita-tive real-time PCR; 16S rRNA, 16S ribosomal RNA; ddPCR, Droplet Digital PCR; TNM, Tumor Node Metastasis.

### Fn infections in cancer

19 studies (Tahara et al., 2014; Ito et al., 2015; Mitsuhashi et al., 2015; Mima et al., 2016; Sun et al., 2016; Park et al., 2017; Yan et al., 2017; Yu et al., 2017; Yamaoka et al., 2018; Chen et al., 2019; Jeong et al., 2019; Kunzmann et al., 2019; Zhang et al., 2019; Boehm et al., 2020; Haruki et al., 2020; Serna et al., 2020; Lee et al., 2021; Nie et al., 2021; Yamamoto et al., 2021) assessed the levels of Fn in cancer tissue samples. The heterogeneity test showed a result of *I*^2^ = 99.0%, indicating a heterogeneity among the studies. Therefore, the random-effect model was chosen. We further analyzed the subgroups according to race (Asian VS. No-Asian). It presented that the analysis results of each subgroup [Asian: *I*^2^ = 98.7%, *P* = 0.001, 0.31(0.29, 0.32); No-Asian: *I*^2^ = 98.8%, *P* = 0.001, 0.09(0.08, 0.10)] were basically consistent with the overall results. Thus, the subgroup analysis did not explain the heterogeneity. To further explore the potential source of the heterogeneity across the studies, we performed sensitivity analyses. First, when the 2 studies (Boehm et al., 2020; Nie et al., 2021) on GC and the studies (Mitsuhashi et al., 2015) on PAAD were excluded, there were still significant heterogeneity [*I*^2^ = 98.8%, *P* = 0.001, 0.21(0.2, 0.23)]. Then, the heterogeneity was still significant after removing each study (Supplementary Table S2). Thus, we refrained from meta-analysis of the Fn prevalence outcome. Across the 19 studies, the incidence of Fn prevalence varied considerably (range: 6.1 to 83.3%) and was greater than 10% in 13 of 19 studies.

### Correlation between the Fn levels and clinicopathological factors in cancer patients (high levels vs. no/low levels)

Three studies (Park et al., 2017; Jeong et al., 2019; Lee et al., 2021) reported the correlation between mucus and the Fn levels in CRC tissues, while 9 studies (Sun et al., 2016; Park et al., 2017; Yan et al., 2017; Yamaoka et al., 2018; Chen et al., 2019; Jeong et al., 2019; Zhang et al., 2019; Boehm et al., 2020; Lee et al., 2021) reported the correlation between the differentiation and Fn levels in the CRC and GC tissues, 2 studies (Park et al., 2017; Lee et al., 2021) reported the correlation between the signet-ring cells and Fn levels in CRC tissues, 5 studies (Mitsuhashi et al., 2015; Park et al., 2017; Boehm et al., 2020; Lee et al., 2021; Nie et al., 2021) reported the correlation between the lymphatic metastasis and Fn levels in cancer tissues, two studies (Park et al., 2017; Nie et al., 2021) reported the correlation between peritumoral lymphocytes and Fn levels in CRC and GC tissues. The results revealed that there seems no statistically significance between the present and absent groups (mucus: *I*^2^ = 0, *P* = 0.54; differentiation: *I*^2^ = 0, *P* = 0.32; signet-ring: *I*^2^ = 0, *P* = 0.71; lymphatic metastasis: *I*^2^ = 0, *P* = 0.43; peritumoral lymphocytes: *I*^2^ = 0, *P* = 0.66). 14 studies (Ito et al., 2015; Mitsuhashi et al., 2015; Mima et al., 2016; Sun et al., 2016; Park et al., 2017; Yamaoka et al., 2018; Chen et al., 2019; Kunzmann et al., 2019; Zhang et al., 2019; Boehm et al., 2020; Haruki et al., 2020; Lee et al., 2021; Nie et al., 2021; Yamamoto et al., 2021) reported the correlation between TNM and Fn levels in CRC tissues. Nine studies (Mima et al., 2016; Sun et al., 2016; Yamaoka et al., 2018; Chen et al., 2019; Jeong et al., 2019; Boehm et al., 2020; Haruki et al., 2020; Lee et al., 2021; Yamamoto et al., 2021) reported the correlation between pN and Fn levels in CRC and GC tissues, the difference was not statistically significant [(III–IV VS. I–II): *p* = 0.12; (pN1/2 VS. pN0): *p* = 0.41] and with high heterogeneity [TNM: *I*^2^ = 53.4; pN: *I*^2^ = 74.1]. We performed a sensitivity analysis to further identify the possible origins of heterogeneity (see sensitivity analysis for details). The above results are provided in Supplementary Figures S1–S7.

**Figure 2:**
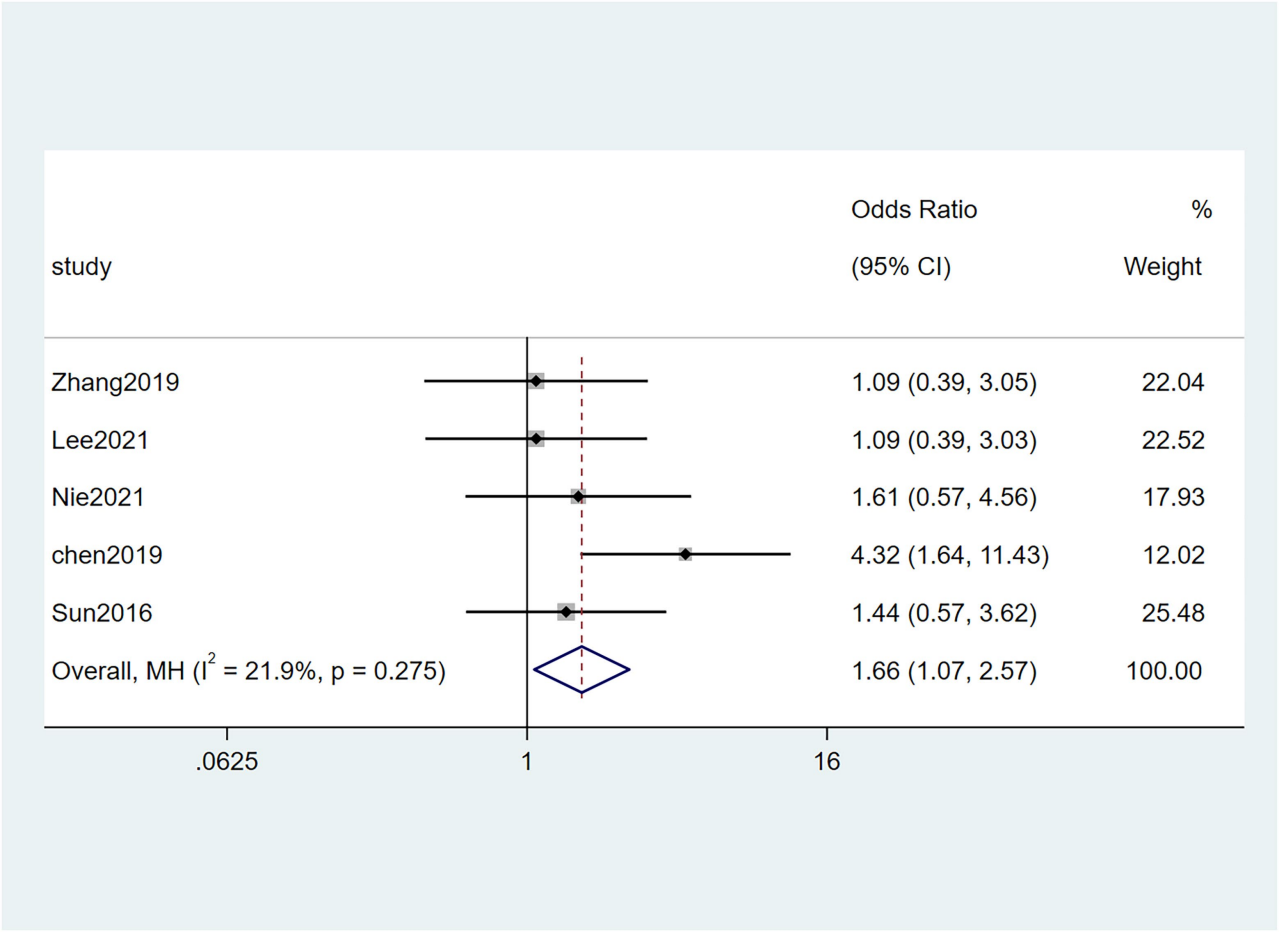
Forest plot of the relationship between *Fusobacterium nucleatum* and vascular invasion in cancer.

**Figure 3:**
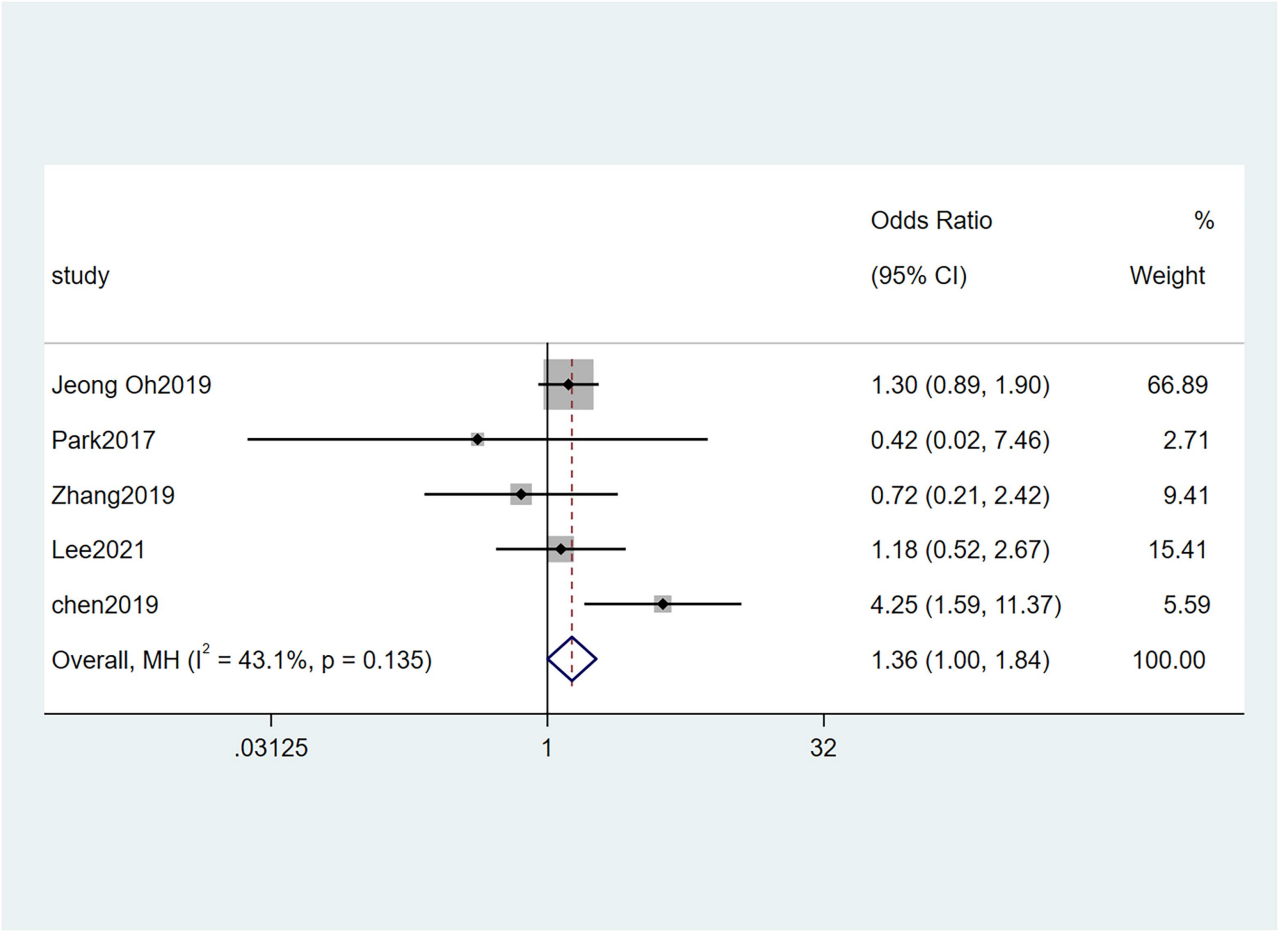
Forest plot of the relationship between *Fusobacterium nucleatum* and nerve invasion in cancer.

**Figure 4:**
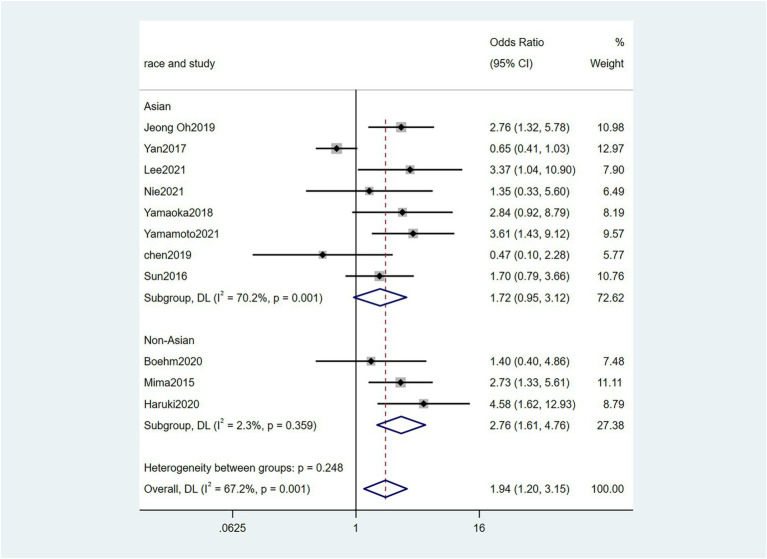
Forest plot of the relationship between *Fusobacterium nucleatum* and infiltration depth(pT) in cancer.

**Figure 5:**
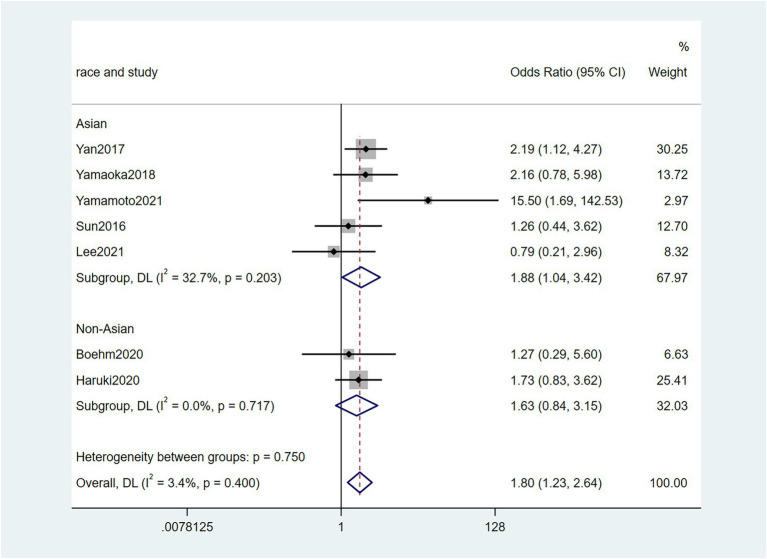
Forest plot of the relationship between *Fusobacterium nucleatum* and distant metastasis(pM) in cancer.

Five studies (Sun et al., 2016; Chen et al., 2019; Zhang et al., 2019; Lee et al., 2021; Nie et al., 2021) reported the correlation between vascular invasion and the Fn levels in CRC tissues, while 5 studies (Park et al., 2017; Chen et al., 2019; Jeong et al., 2019; Zhang et al., 2019; Lee et al., 2021) reported the correlation between nerve invasion and Fn levels in CRC and GC tissues, 11 studies (Mima et al., 2016; Sun et al., 2016; Yan et al., 2017; Yamaoka et al., 2018; Chen et al., 2019; Jeong et al., 2019; Boehm et al., 2020; Haruki et al., 2020; Lee et al., 2021; Nie et al., 2021; Yamamoto et al., 2021) reported the correlation between infiltration depth (pT)and Fn levels in CRC and GC tissues, 7 studies (Sun et al., 2016; Yan et al., 2017; Yamaoka et al., 2018; Boehm et al., 2020; Haruki et al., 2020; Lee et al., 2021; Yamamoto et al., 2021) reported the correlation between distant metastasis (pM)and Fn levels in CRC and GC tissues. The difference between the two groups was statistically significant[vascular invasion (present VS. absent): *P* = 0.02; nerve invasion (present VS. absent): *P* = 0.04; pT(T3/4 VS. T1/2): *P* = 0.01; pM(M1 VS. M0): *P* = 0.01]. Furthermore, the present vascular invasion group showed high Fn levels compared to the absent group [OR = 1.66, 95%CI(1.07, 2.57), *I*^2^ = 21.9%, fixed effect model, Figure 2]. The present nerve invasion group showed a high Fn level compared to the absent group [OR = 1.36, 95%CI(1.00, 1.84), *I*^2^ = 43.1%, fixed effect model, Figure 3]. Higher Fn levels were observed in the pT3/4 group compared to the pT1/2 group [OR = 1.73, 95%CI(1.36, 2.21), *I*^2^ = 67.4%, random effect model, Figure 4]. Due to the existence of significant heterogeneity (*I*^2^ = 67.4%), we further performed the analyses to identify potential sources of heterogeneity (see sensitivity analysis for details). Higher Fn levels were observed in the pM1 group compared to pM0 group [pM: OR = 1.84, 95%CI(1.27, 2.65), *I*^2^ = 3.6%, fixed effect model, Figure 5]. The detailed results are shown in Table 2.

**Table 2:** The correlation between *Fusobacterium nucleatum* and the clinicopathological features of colorectal cancer.

Clinicopathology	Studies	Participants	OR, 95%CI	*p*-value	Heterogeneity	Analysis
					(*I*^2^,*p*-value)	
Mucus	3	879	1.40(0.74, 1.78)	0.54	0%, 0.73	Fix
Differentiation	10	2,732	1.43(1.10, 1.87)	0.07	51.8%, 0.78	Random
Signet-ring cells	2	286	1.18(0.49, 2.88)	0.71	0%, 0.62	Fix
Lymphatic metastasis	5	1,223	1.12(0.85, 1.47)	0.43	0%, 0.89	Fix
Vascular invasion	5	524	1.66(1.07, 2.57)	0.02	21.9%, 0.28	Fix
Nerve invasion	5	1,064	1.36(1.00, 1.84)	0.04	43.1%, 0.14	Fix
Peridumoral lymphocytes	2	219	1.19(0.56, 2.53)	0.66	0%, 0.01	Fix
pT	11	3,206	1.94(1.20, 3.15)	0.01	67.2%, 0.01	Random
Asian	8	1,475	1.72(0.95, 3.12)	0.07	70.2%, 0.01	
Non-Asian	3	1731	2.76(1.61, 4.76)	0.01	2.3%, 0.36	
pN	9	2,946	1.28(1.02, 1.61)	0.41	74.1%, 0.01	Random
pM	7	1,606	1.84(1.27, 2.65)	0.01	3.6%, 0.40	Fix
Asian	5	858	1.88(1.04, 3.42)	0.04	32.7%, 0.20	
Non-Asian	2	748	1.63(0.84, 3.15)	0.15	0%, 0.72	
TNM	14	3,575	1.15(0.95, 1.39)	0.15	53.4%,0.73	Random

TNM, Tumor Node Metastasis; pT, infiltration depth; pN, regional lymph node metastasis; pM, distant metastasis; CI, confidence interval.

### Association between the Fn levels and molecular characteristics of cancer patients

**Figure 6:**
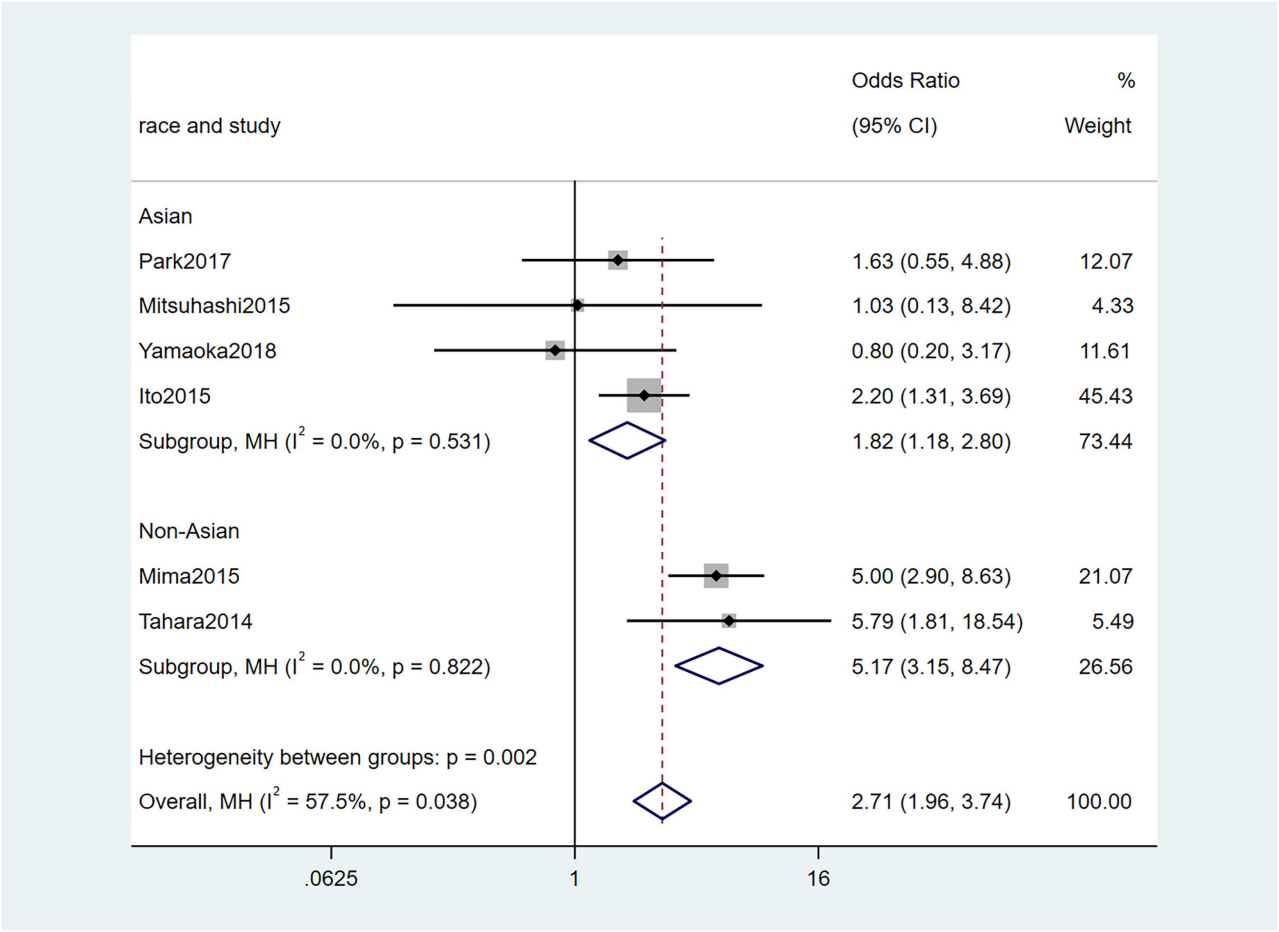
Forest plot of the relationship between *Fusobacterium nucleatum* and MLH1 methylation in cancer.

**Figure 7:**
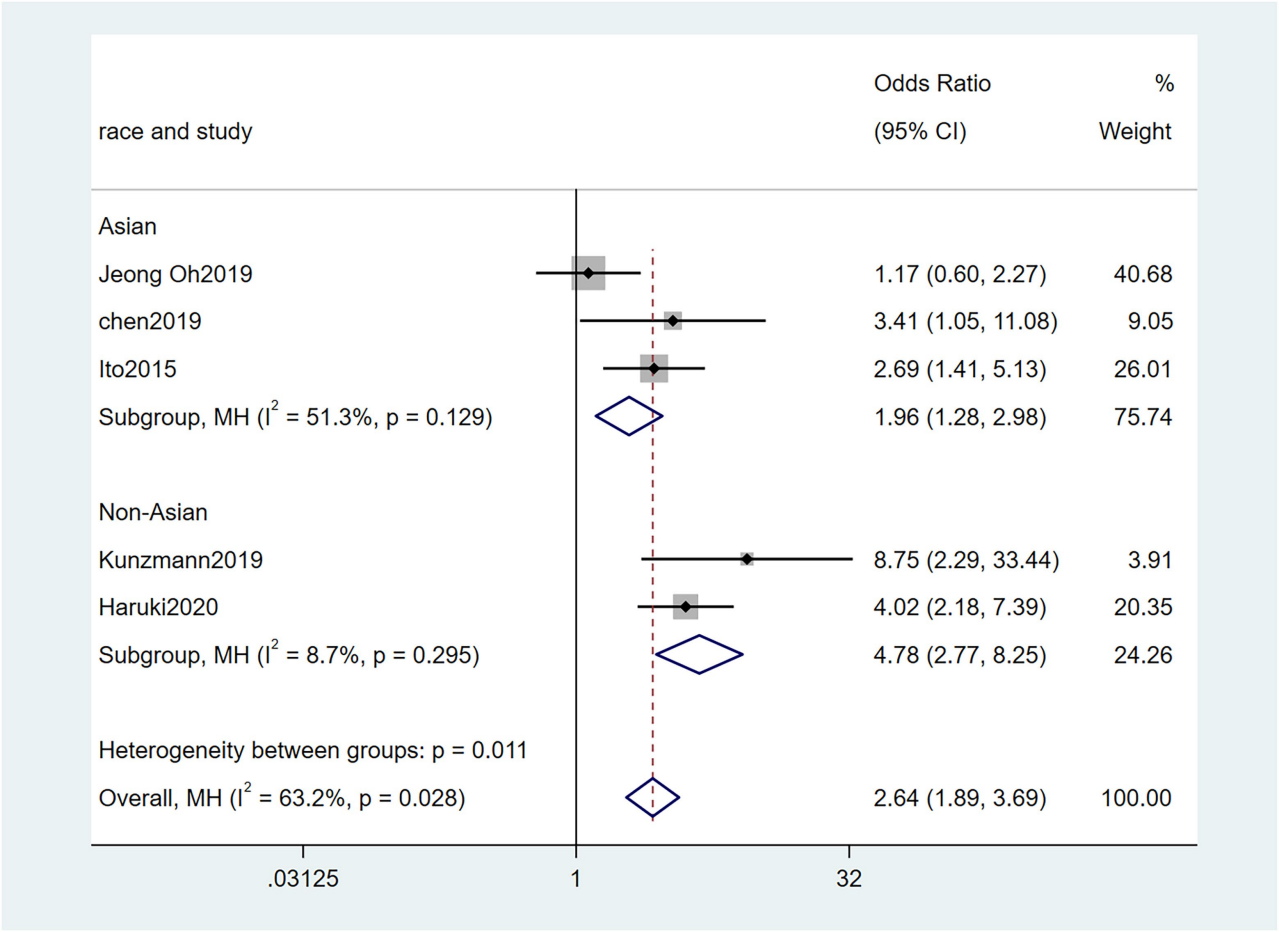
Forest plot of the relationship between *Fusobacterium nucleatum* and MSI status in cancer.

**Figure 8:**
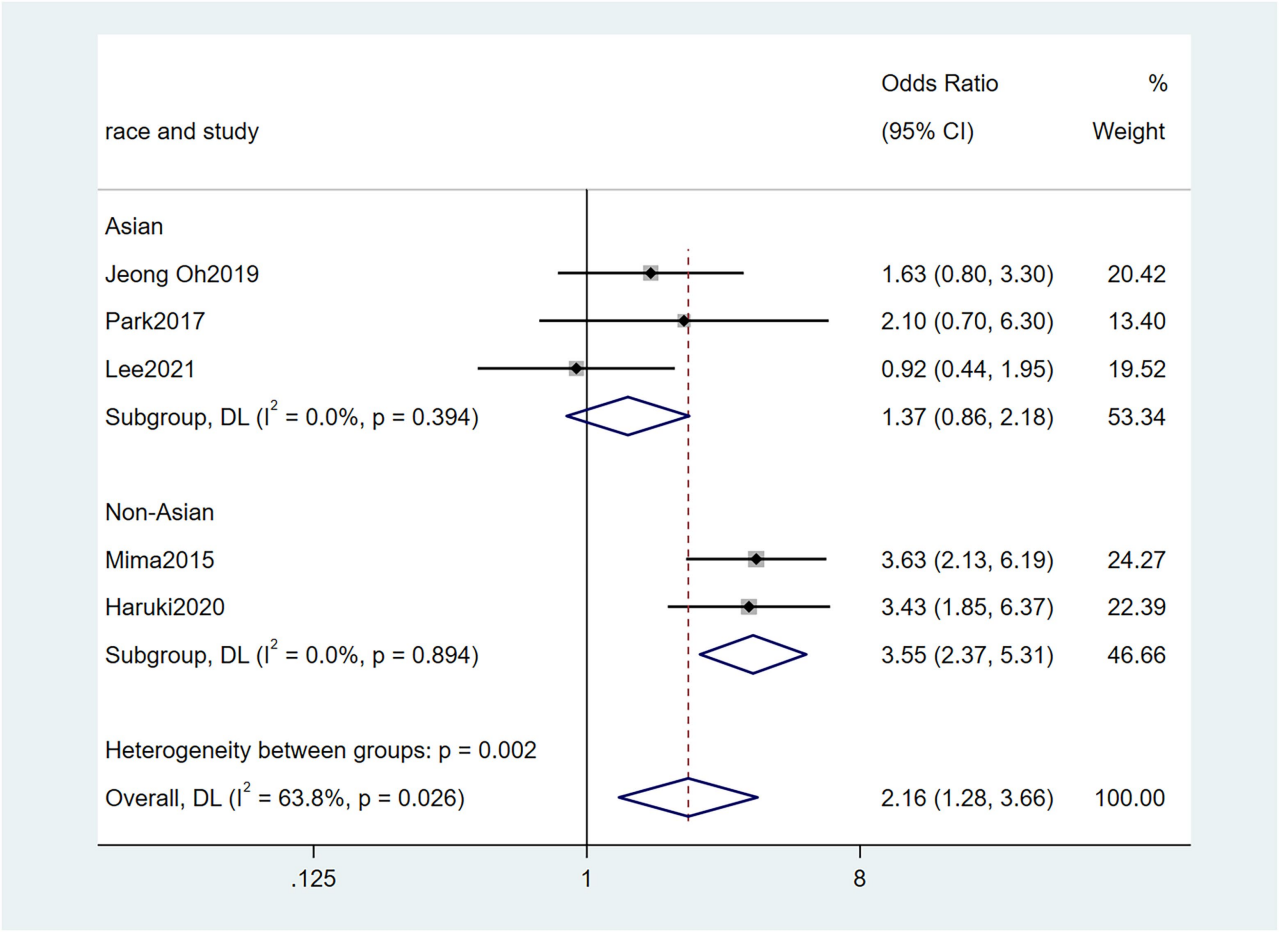
Forest plot of the relationship between *Fusobacterium nucleatum* and CIMP methylation in cancer.

**Figure 9:**
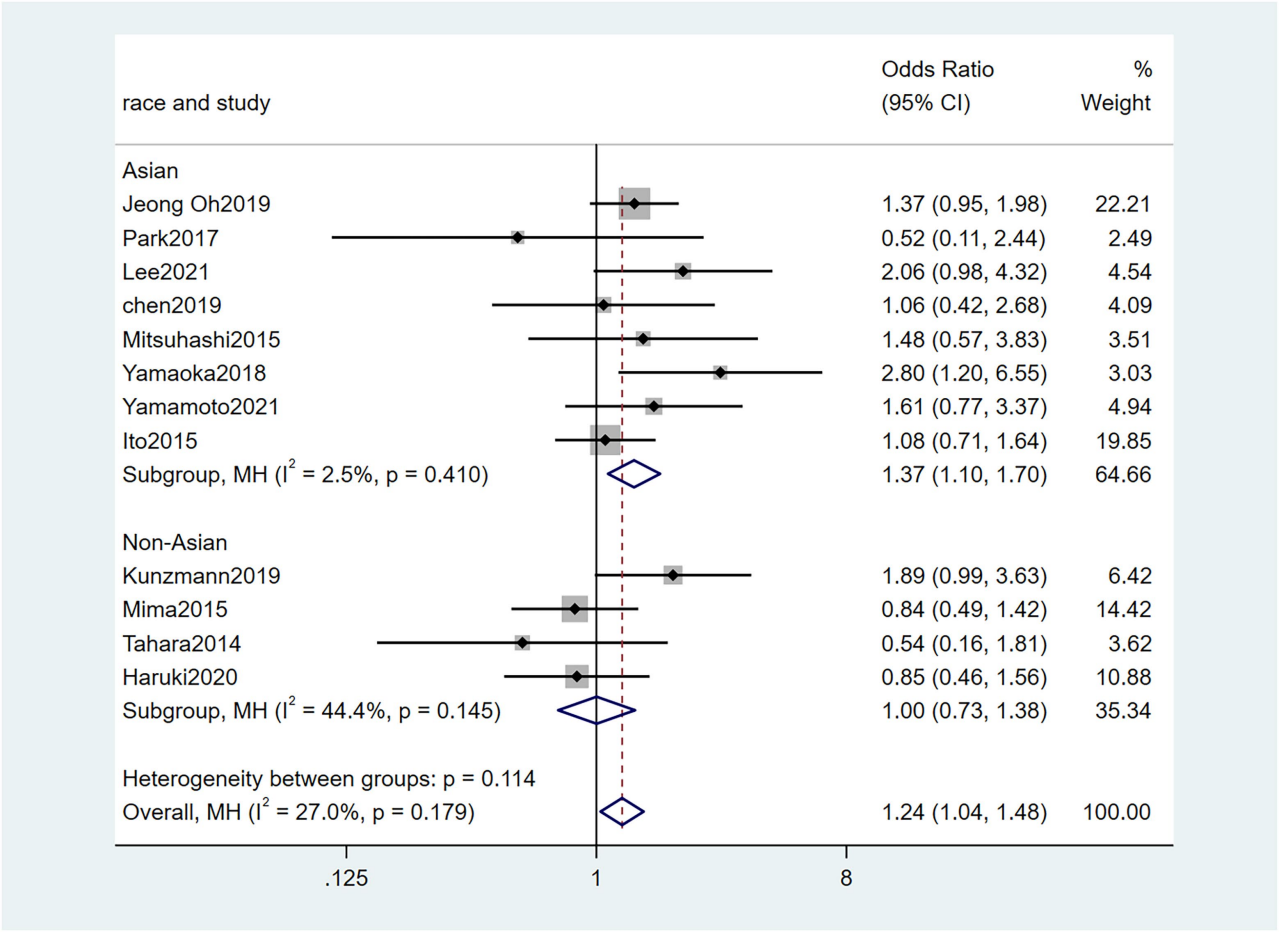
Forest plot of the relationship between *Fusobacterium nucleatum* and KRAS mutation in cancer.

**Figure 10:**
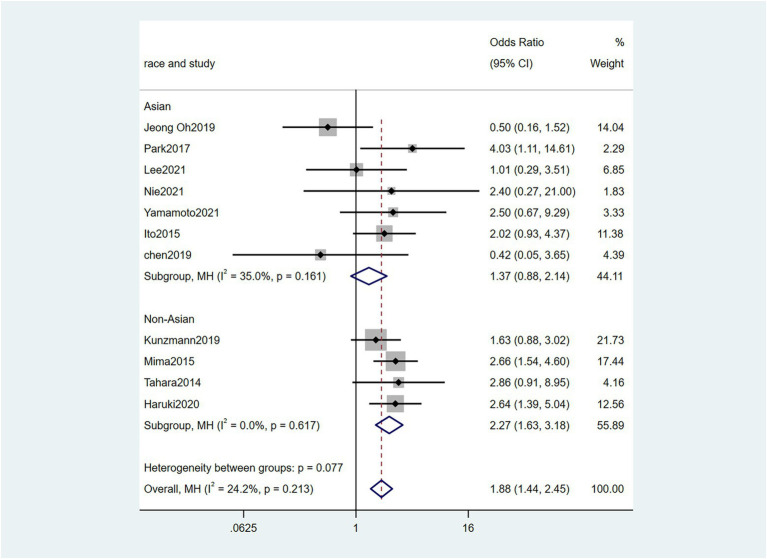
Forest plot of the relationship between *Fusobacterium nucleatum* and BRAF mutation in cancer.

To further investigate the effect of Fn levels in cancer, we extracted the molecular characteristic data from each study. 6 studies (Tahara et al., 2014; Ito et al., 2015; Mitsuhashi et al., 2015; Mima et al., 2016; Park et al., 2017; Yamaoka et al., 2018) were analyzed for the association between the Fn levels and MLH1 methylation status. The pooled results showed that patients with MLH1 methylation usually had a higher Fn level than those in the unmethylated group [OR = 2.53, 95%CI(1.42, 4.53), *P* = 0.01, *I*^2^ = 57.5%, random effect model, subgroups (Asian: *P* = 0.01, *I*^2^ = 0%; Non-Asian: *P* = 0.01, *I*^2^ = 0%), Figure 6]. Patients with MSI-high had the higher Fn levels compared to those with MSS/MSI-low from 5 studies(Ito et al., 2015; Chen et al., 2019; Jeong et al., 2019; Kunzmann et al., 2019; Haruki et al., 2020) [OR = 2.92, 95%CI(1.61, 5.32), *P* = 0.01, *I*^2^ = 63.2%, random effect model, subgroups (Asian: *P* = 0.01, *I*^2^ = 51.3%; Non-Asian: *P* = 0.01, *I*^2^ = 8.7%), Figure 7]. The pool of data from 6 studies (Tahara et al., 2014; Ito et al., 2015; Mitsuhashi et al., 2015; Mima et al., 2016; Park et al., 2017; Yamaoka et al., 2018) showed that a high level CIMP was positively correlated with the high levels of Fn [OR = 2.23, 95%CI(1.64, 3.03), *P* = 0.01, *I*^2^ = 64.2%, random effect model, subgroups (Asian: *P* = 0.19, *I*^2^ = 0%; Non-Asian: *P* = 0.01, *I*^2^ = 0%), Figure 8]. Higher Fn levels were observed in the KRAS mutation group compared to the wild group from 12 studies (Tahara et al., 2014; Ito et al., 2015; Mitsuhashi et al., 2015; Mima et al., 2016; Park et al., 2017; Yamaoka et al., 2018; Chen et al., 2019; Jeong et al., 2019; Kunzmann et al., 2019; Haruki et al., 2020; Lee et al., 2021; Yamamoto et al., 2021) [OR = 1.24, 95%CI(1.04, 1.48), *P* = 0.02, *I*^2^ = 27.0%, fixed effect model, Asian: *P* = 0.01, *I*^2^ = 2.5%; Non-Asian: *P* = 0.99, *I*^2^ = 44.4%), Figure 9]. Similarly, the pooled results of 11 studies (Tahara et al., 2014; Ito et al., 2015; Mima et al., 2016; Park et al., 2017; Chen et al., 2019; Jeong et al., 2019; Kunzmann et al., 2019; Haruki et al., 2020; Lee et al., 2021; Nie et al., 2021; Yamamoto et al., 2021) showed that BRAF mutation was positively associated with Fn levels [OR = 1.88, 95%CI(1.44, 2.45), *P* = 0.01, *I*^2^ = 24.2%, fixed effect model, Asian: *P* = 0.16, *I*^2^ = 35%; Non-Asian: *P* = 0.01, *I*^2^ = 0%), Figure 10]. The details of the above results are provided in Table 3.

**Table 3:** The correlation between *Fusobacterium nucleatum* and molecular characteristics of cancer.

Genetic mutations	Studies	Participants	OR 95%CI	p-value	Heterogeneity	Analysis
					(*I*^2^, *p*-value)	
MLH1methylation	6	2,144	2.53(1.42, 4.53)	0.01	57.5%, 0.04	Random
Asian	4	1,017	1.84(1.19, 2.84)	0.01	0%, 0.53	
Non-Asian	2	1,127	5.14(3.14, 8.42)	0.01	0%, 0.82	
MSI	5	2049	2.92(1.61, 5.32)	0.01	63.2%, 0.03	Random
Asian	3	1,187	1.96(1.28, 2.98)	0.01	51.3%, 0.13	
Non-Asian	2	862	4.78(2.77, 8.26)	0.01	8.7%, 0.30	
CIMP methylation	5	2,519	2.23(1.64, 3.03)	0.01	64.2%, 0.03	Random
Asian	3	877	1.37(0.86, 2.18)	0.19	0%, 0.39	
Non-Asian	2	1,642	3.55(2.37, 5.31)	0.01	0%, 0.89	
KRAS mutation	12	4,084	1.24(1.04, 1.48)	0.02	27.0%, 0.18	Fix
Asian	8	2054	1.36(0.71, 1.64)	0.01	2.5%, 0.41	
Non-Asian	4	2030	1.00(0.73, 1.38)	0.99	44.4%, 0.15	
BRAF mutation	11	3,841	1.88(1.44, 2.45)	0.01	24.2%, 0.21	Fix
Asian	7	1770	1.38(0.88, 2.15)	0.16	35.0%, 0.16	
Non-Asian	4	2071	2.72(1.63, 3.18)	0.01	0%, 0.62	

CI, confidence interval.

### Correlation between Fn levels and relapse-free survival in CRC patients

**Figure 11:**
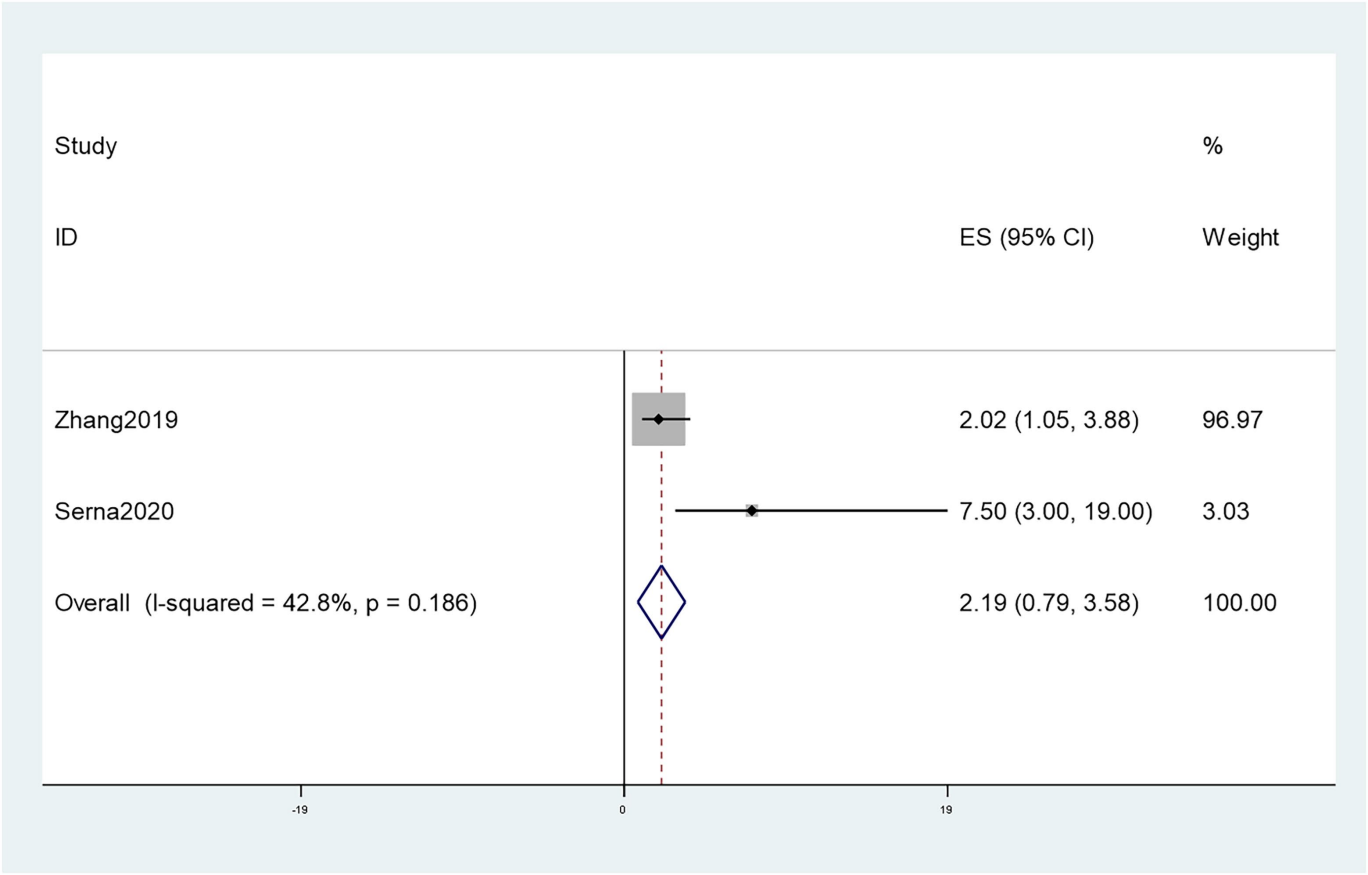
Forest plot of the relationship between *Fusobacterium nucleatum* and RFS in cancer.

Two studies (Zhang et al., 2019; Serna et al., 2020) reported the association between the RFS and Fn levels. Our results demonstrated that the CRC patients with high levels of Fn had a worse RFS than those with no/low levels of Fn [HR = 2.19, 95%CI(0.79, 3.58), *P* = 0.01, *I*^2^ = 42.8%, random effect model, shown in Figure 11].

### Sensitivity and subgroup analysis

For the comparisons with significant heterogeneity, we performed a sensitivity analysis in this meta-analysis. The sensitivity analysis was performed by omitting individual studies one by one to assess their effect on the aggregated results. When Yamamoto et al. (2021) was removed, the heterogeneity of pN directly decreased to 0%. The heterogeneity of pT was high (*I*^2^ = 67.4%), but there was no heterogeneity after removing the study of Yan et al. (2017) (*I*^2^ = 1.8%). The heterogeneity of MLH1 methylation was high (*I*^2^ = 57.5%), and the heterogeneity was *I*^2^ = 27% after the study of Mima et al. (2016) was removed. In addition, the heterogeneity of MSI status results was high (*I*^2^ = 63.2%). when the study of Jeong et al. (2019) was removed, the heterogeneity decreased (*I*^2^ = 0%). Heterogeneity in sensitivity analysis of CIMP status results remained high after each study was omitted sequentially. All results are provided in Supplementary Table S2.

To further clarify the heterogeneity source, subgroup analysis was performed according to race, and the results of MLH1 methylation, CIMP status indicated that race was the main source of heterogeneity. However, the results of pT, pN, and MSI status showed the heterogeneity did not decrease in the subgroup analyses of both Asian and No-Asian.

## Risk of bias

In this meta-analysis, three corresponding methods were used to evaluate publication bias: Begg’s adjusted rank correlation test, and Egger’s regression test. Statistical significance was also not observed according to Egger’s and Begg’s tests (Egger et al., 2021; *P* > 0.05; Table 4). There was no publication bias in our included studies. However, according to the limited number of literatures, the presence of a publication bias cannot be conclusively excluded.

**Table 4:** Risk of bias for meta-analysis.

	Begg		Egger		
Factor	Z	*P*	95%CI	*t*	*P*
Vascular invasion	0.24	0.81	−97.12, 54.72	−0.89	0.44
Nerve invasion	0	1	−52.38, 57.34	0.58	0.67
pT	1.07	0.28	−4.43, 1.16	−1.35	0.21
pM	0.30	0.76	−2.75, 3.58	0.34	0.75
MLH1 methylation	0.01	1	−2.53, 5.30	0.99	0.38
MSI state	0.24	0.81	−6.22, 10.65	0.84	0.47
CIMP methylation	0.12	0.90	−5.81, 6.25	0.09	0.93
KRAS mutation	−0.07	1.00	−2.04, 2.22	0.09	0.93
BRAF mutation	0.41	0.68	−1.82, 2.70	0.43	0.68

## Discussion

Recently, increasing evidence suggests an important association between the Fn levels and CRC. Here, we performed a more comprehensive and detailed meta-analysis (Huangfu et al., 2021) on this association. To explore the correlation between the Fn infections and cancers more comprehensively, the scope of the literature search was not limited to CRC. Unfortunately, only 2 studies on GC and 1 study on PAAD passed the inclusion criteria. More research on Fn infections across pan-cancer should be considered in future.

Among the existing studies, Li et al. (2016) investigated 101 Chinese patients with CRC and reported that the Fn infection rate was as high as 87.1%. Another study (Mima et al., 2015) analyzed 598 CRC cases by qPCR, which found an Fn infection rate of 13.0% in tumor tissues. Nie et al. (2021) and Boehm et al. (2020) observed the Fn positivity in GC patients in 28.8 and 32.75%, respectively. The detection rate of Fusobacterium species in PAAD tissue specimens in the Mitsuhashi et al. (2015) was 8.8%. A report (Nosho et al., 2016) from Japan presented that the positive rate of Fn was 8.6%. Thus, no meta-analysis could be conducted due to the considerable heterogeneity in our study. Across these 19 studies, the incidence of Fn prevalence varied considerably (range: 6.1 to 83.3%) and was greater than 10% in 13 of 19 studies. The high heterogeneity observed could be due to variation in study design and/or population. However, as can be seen, Fn infections are already prevalent in cancer.

Our results indicated that the high Fn levels were significantly correlated with the presence of vascular and nerve invasion, suggesting that Fn may promote the aggressive potential of tumor cells. On the other hand, Lee et al. (2021) reported that vascular, and nerve invasion were not related to Fn levels (*P* > 0.05). This conclusion might be conducted due to a small corhort (Lee et al., 2021), compared to other research, among which the number of advanced cancer was only 36 cases. To our best knowledge, meta-analyse of the high Fn levels and vascular, nerve invasion has not been previously reported. As one of the main approaches to the pM1 of CRC, nerve invasion is closely related to the depth of tumor invasion and lymph node metastasis, which may affect postoperative tumor recurrence and metastasis to a certain extent and also reflect the prognosis of patients (Liebl et al., 2013; Ueno et al., 2014). Besides, we extracted the data on the correlation among pT, pN, pM, and Fn levels, and the results clearly showed that the high Fn levels were positively correlated with poor pathological status (pT3/4; pM1). Heterogeneity regarding the pooled data for pN was high (*I*^2^ = 74.1%), although the results seemed not statistically significant by a random-effect model. The main source of heterogeneity was introduced by the work of Yamamoto et al. (2021). In this study, the number of pN1/2 cases accounted for 10% of the total number of cases and that of other studies were in the range of 26–90%. These results are in excellent agreement with the published meta-analyses (Huangfu et al., 2021). A study (Casasanta et al., 2020) has shown that Fn directly induced metastasis by releasing cytokines, increasing NF-κB expression, and subsequently expressing KRT7 (Chen et al., 2020), increasing CARD3, and downregulating E-cadherin (Chen et al., 2020). Notably, another study has shown that Fn activates the β-catenin signaling pathway in CRC through LPS mediated Toll-like receptor 4 (TLR4)/ p21-activated kinase 1 (PAK1; Chen et al., 2017). TLR4 activates the β-catenin signaling pathway and forms intestinal tumors, while PAK1 is associated with CRC progression and metastasis (Wu et al., 2018). Mekenkamp et al. (2012) found that the presence of mucus in cancers indicates resistance to chemotherapy and also implies a worse prognosis for cancer patients. Multiple studies have shown that the signet ring cell carcinoma of CRC is often associated with lymph node metastasis, even if it is treated with radical resection, which is associated with poor prognosis (Mizushima et al., 2010; Hartman et al., 2013). Thus, to obtain a comprehensive study, we included signet ring cells and mucus factor, and the combined results showed no statistically significant difference.

In our study, the high Fn levels of cancer tissue were associated with key tumor molecular features of CRC, including MLH1 methylation, MSI-high, CIMP-high, KRAS mutation, and BRAF mutation, which were associated with clinical outcomes in CRC. The results of Jeong et al. (2019) considered no correlations between these molecular characteristics and Fn levels. A meta-analyse (Huangfu et al., 2021) also suggested no association between KRAS mutation and Fn levels. However, CRC arises from the transformation of normal mucosa to precancerous lesions, and colorectal adenoma progresses to cancer over several years. In the serrated polyp pathway, the genetic changes involved BRAF and KRAS mutation. Both KRAS and BRAF encode kinases belonging to the mitogen-activated protein kinase (MAPK) cascade that mediate cell signaling involved in cell proliferation, apoptosis, and differentiation (Yamane et al., 2014). Another molecular change is the MSI in serrated lesions, which is attributed to the loss of mismatch repair genes, often leading to increased susceptibility to the accumulation of gene mutations in regions containing microsatellites (Mizushima et al., 2010). MSI status has been evaluated as a prognostic and chemotherapy or immunotherapy response biomarker in CRC patients (Diao et al., 2021). Previous a report suggested that Fn was hypothesized to directly or indirectly interact with MSI-H status (Gao et al., 2021). A series of experimental studies have revealed that Fn may not be a mere passenger colorectal carcinogenesis and would play an active role in tumorigenesisi of MSI-H CRC (Kostic et al., 2013). Heterogeneity was substantial in the MSI status of the present meta-analysis, and the study of Jeong et al. (2019) was the primary source of heterogeneity. The samples in this study come from the patients diagnosed with stage II/III CRC, that may be a reason for its heterogeneity. There was a strong correlation between CIMP and serrated CRC. CIMP-high status as a potential biomarker to predict irinotecan-based chemotherapy regimens for CRC (Vilar and Gruber, 2010; Zhang et al., 2021). Results from subgroup analysis in the current study on MLH1 methylation and CIMP status suggested that race was a potential source of heterogeneity of the pooled results.

Previous meta-analyse (Huangfu et al., 2021) has shown that the high levels of Fn are strongly associated with poor outcomes in patients with CRC, including overall survival, disease-free survival, and cancer-specific survival. We also extracted the data on patients’ RFS and showed that CRC patients with high levels of Fn had worse RFS than patients with no/low Fn levels. Due to a lack of original data for some studies, HRs were extracted from the Kaplan–Meier survival curves, which may affect the accuracy of the results.

In conclusion, our study results show that the Fn could be quite useful to predict unfavorable prognosis and function as potential prognostic biomarkers in CRC. There is a discrepancy between our research and previous studies. Although Jeong et al. (2019) showed that the tumors with high levels of Fn had a better prognosis than those with low or negative levels of Fn in non-sigmoid colon cancers, the difference may be derived from the nature of study population. Their study samples were a well-selected and relatively-homogeneous cohort that contained only stage III or high-risk stage II CRCs treated with oxaliplatin-based adjuvant chemotherapy.

No doubt, certain limitations do exist in this study. We may miss some eligible studies published in other languages, while the unpublished data were not identified. In this case, the publication bias always cannot be absolutely excluded, although no significant publication bias was detected. In addition, there are still many unknowns in the study of bacteria and tumors. For example, whether a bacterial infection is a cause or effect has not been confirmed yet. The existing study (Nejman et al., 2020) has reported that bacteria exist in cancer tissues. We suggest the following questions for further research. How exactly do bacteria colonize tumor tissue? Whether it is because the bacteria invade the vascular epithelium and then the blood metastases to the cancer tissue, or whether the changes in the tumor microenvironment attract the colonization of the bacteria?

## Author contributions

JW and YiW conceived and designed the study. YuW and XuZ evaluated study quality. Study tabulations were carried out by YiW and XL, with final review by XiZ, YX, CR, and YuW performed data analyses. YiW and YH drafted the manuscript. All authors contributed to the article and approved the submitted version.

## Funding

This research was funded by the Natural Science Basic Research Program of Shaanxi, grant number 2020JC-01. The article processing charge was funded by the Natural Science Basic Research Program of Shaanxi, grant number 2020JC-01.

## Conflict of interest

The authors declare that the research was conducted in the absence of any commercial or financial relationships that could be construed as a potential conflict of interest.

## Publisher’s note

All claims expressed in this article are solely those of the authors and do not necessarily represent those of their affiliated organizations, or those of the publisher, the editors and the reviewers. Any product that may be evaluated in this article, or claim that may be made by its manufacturer, is not guaranteed or endorsed by the publisher.

## Supplementary material

The Supplementary material for this article can be found online at: https://www.frontiersin.org/articles/10.3389/fmicb.2022.945463/full#supplementary-material
