# Supplementary figures and images for: Clinicopathological differences of high Fusobacterium nucleatum levels in colorectal cancer: A review and meta-analysis
Source: Front Microbiol. 2022 Nov 4;13:945463. doi: 10.3389/fmicb.2022.945463 (PMC9672069; doi:10.3389/fmicb.2022.945463)

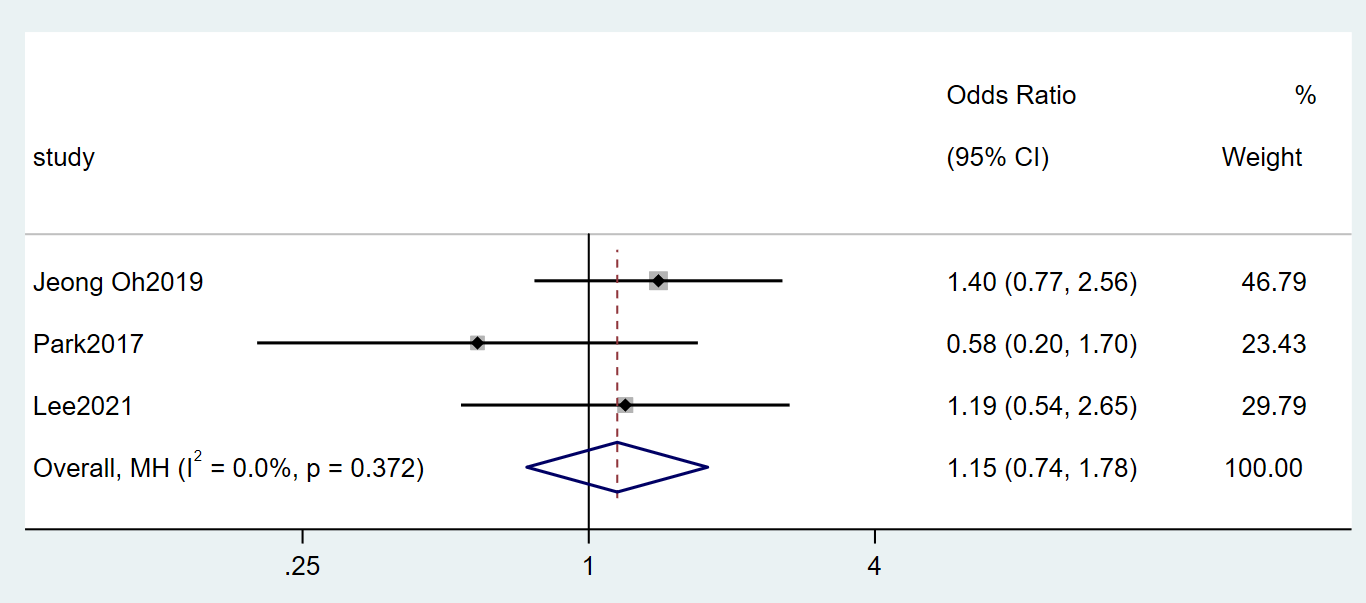

Supplement: Supplementary file 3 [file Image_1.TIF]

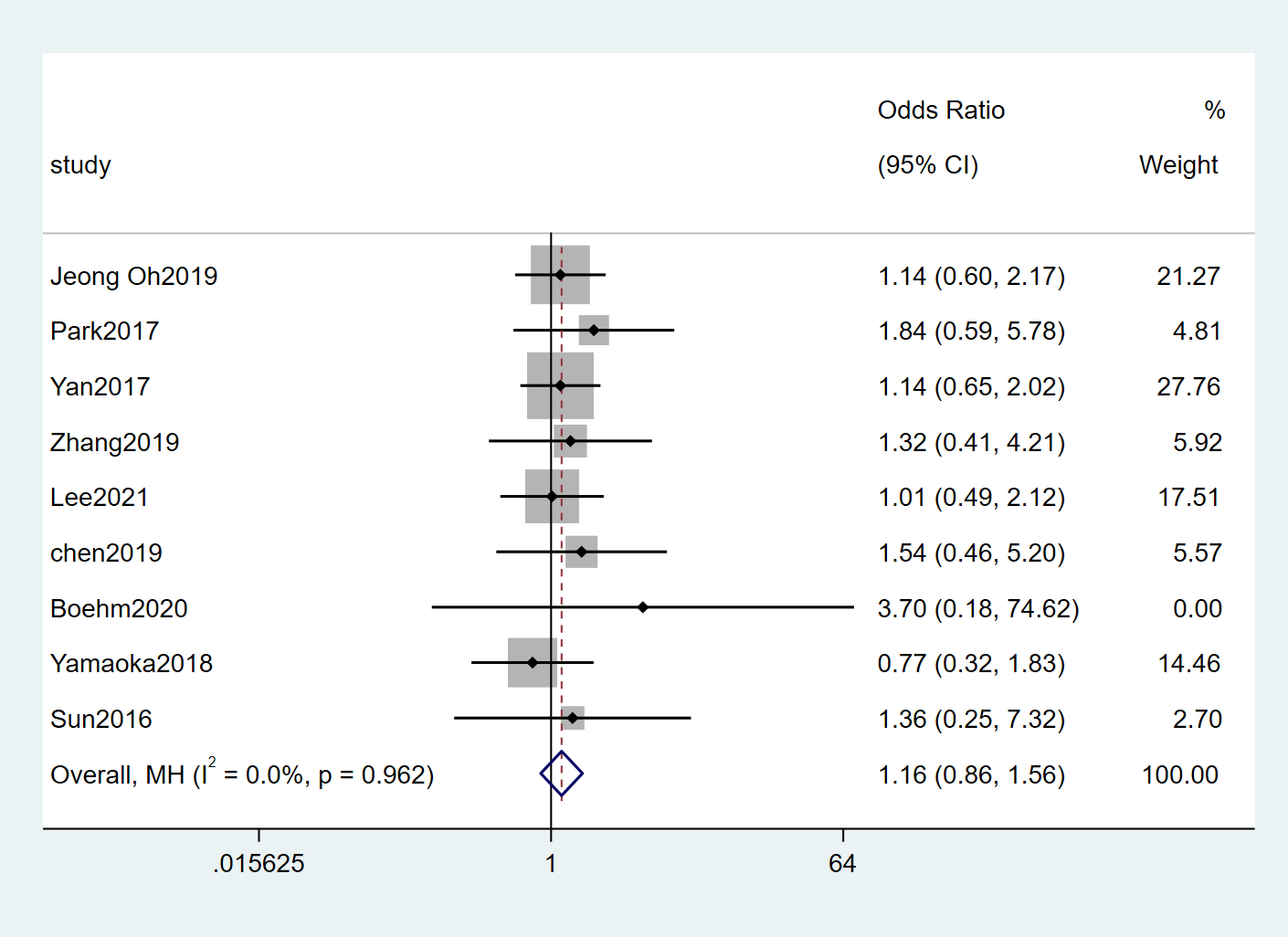

Supplement: Supplementary file 4 [file Image_2.TIF]

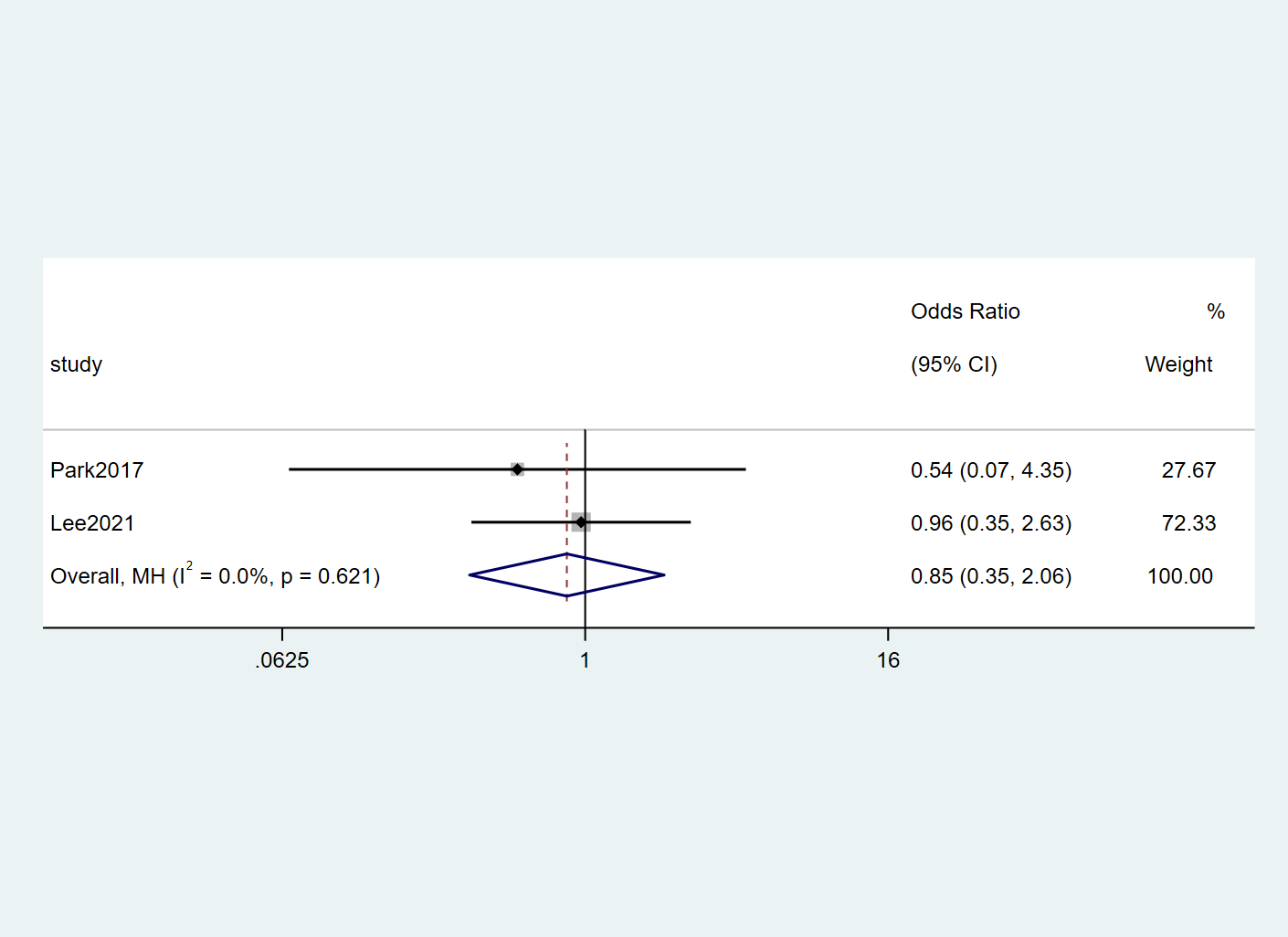

Supplement: Supplementary file 5 [file Image_3.TIF]

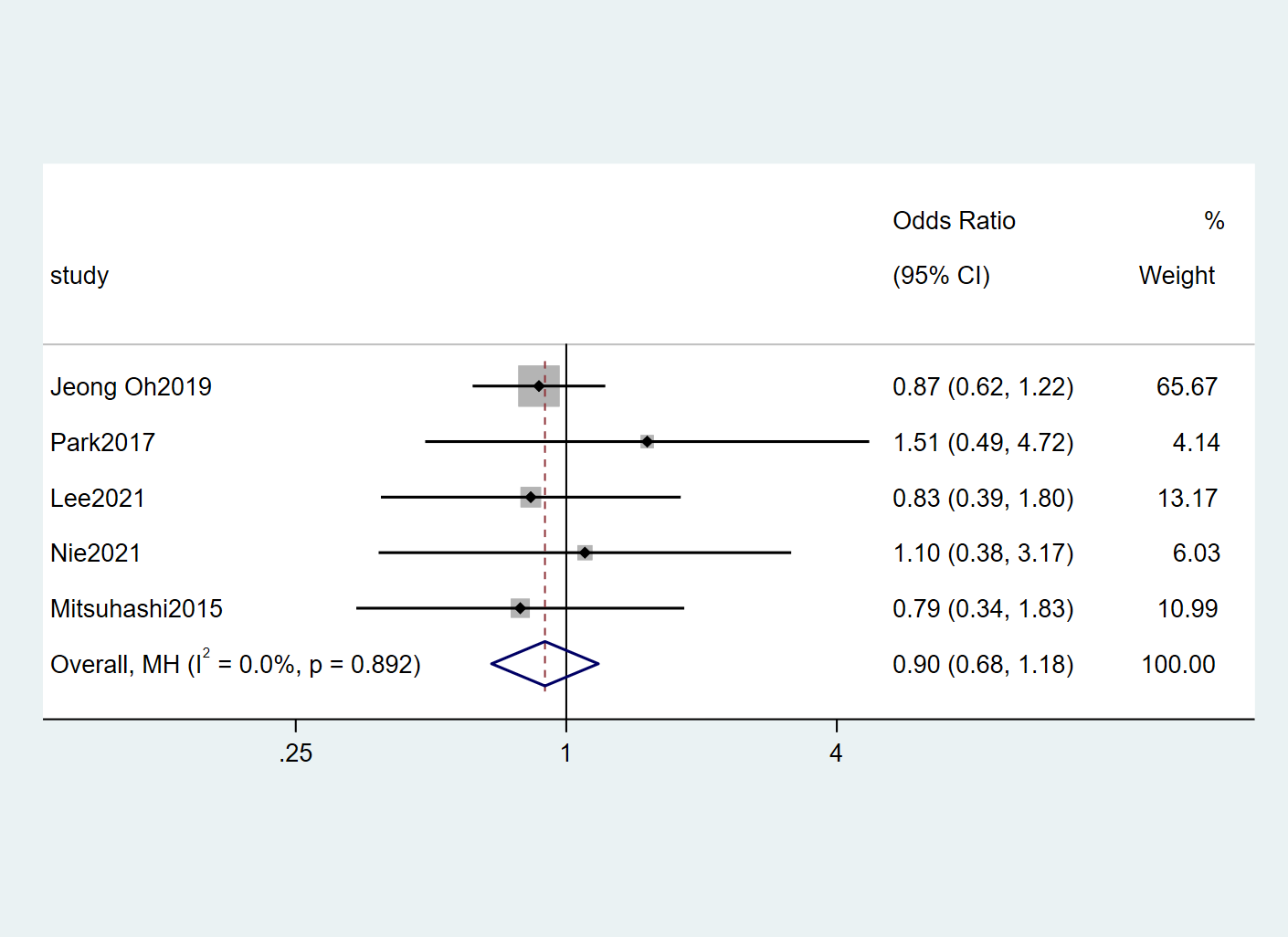

Supplement: Supplementary file 6 [file Image_4.TIF]

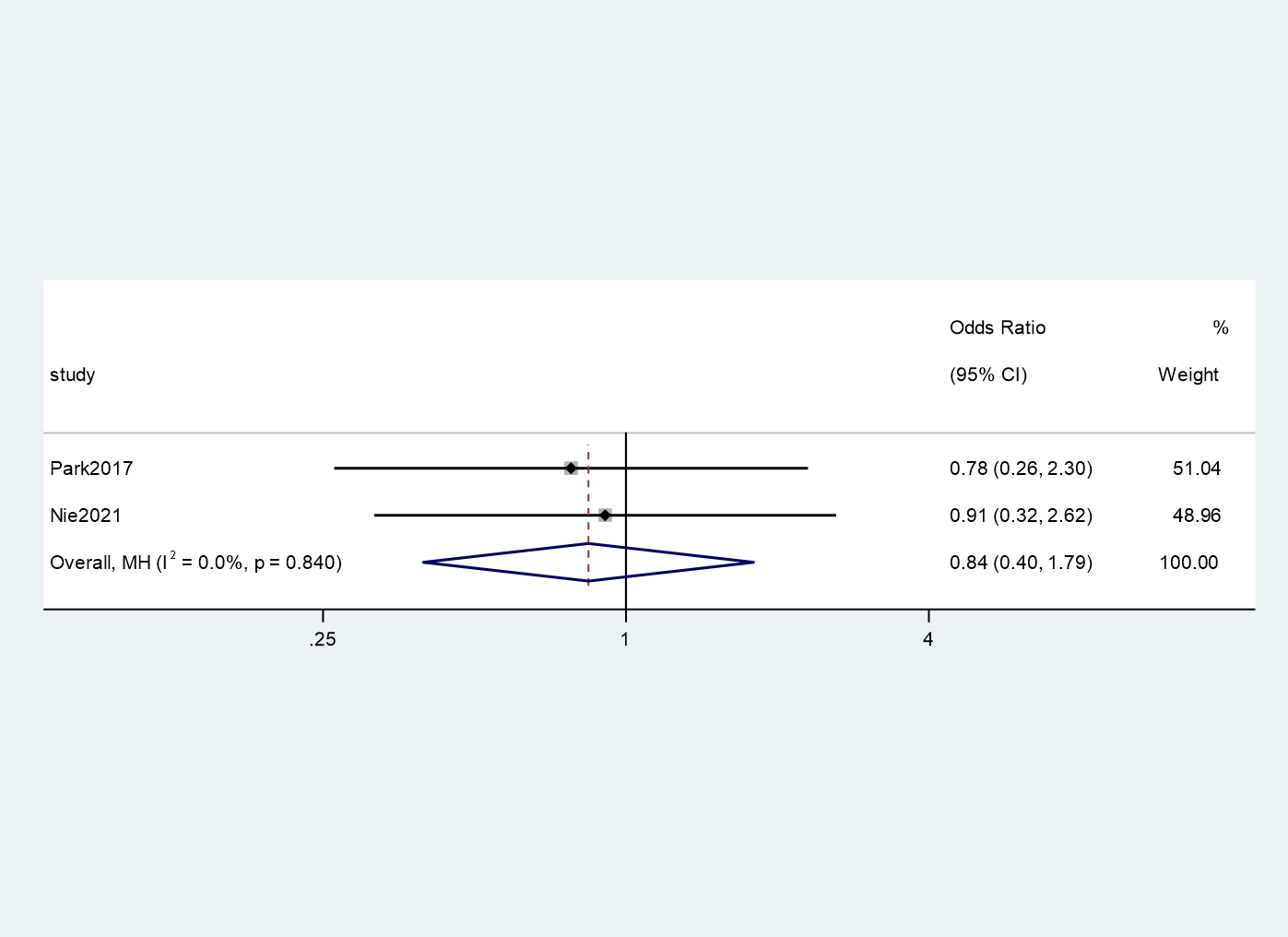

Supplement: Supplementary file 7 [file Image_5.TIF]

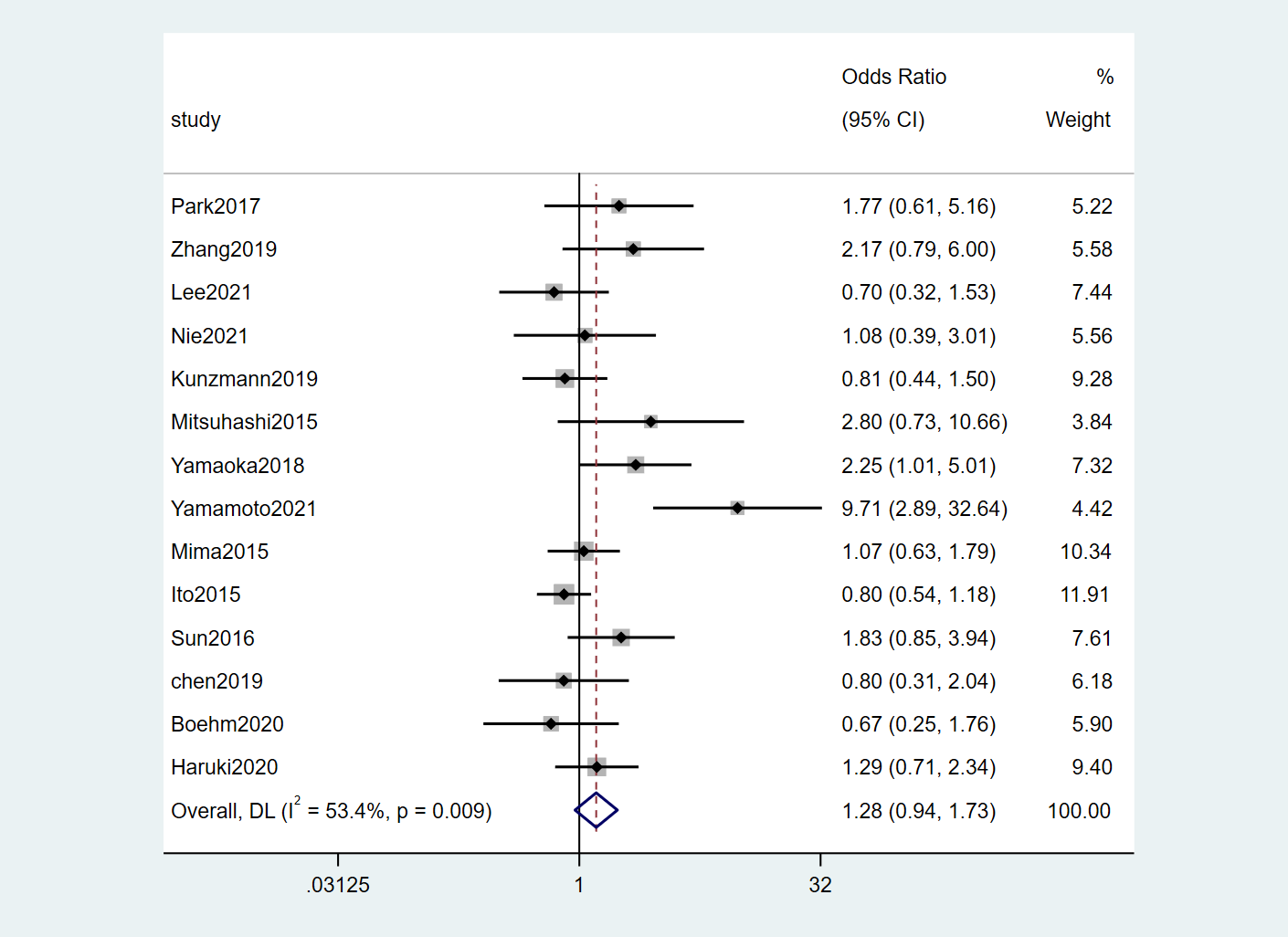

Supplement: Supplementary file 8 [file Image_6.TIF]

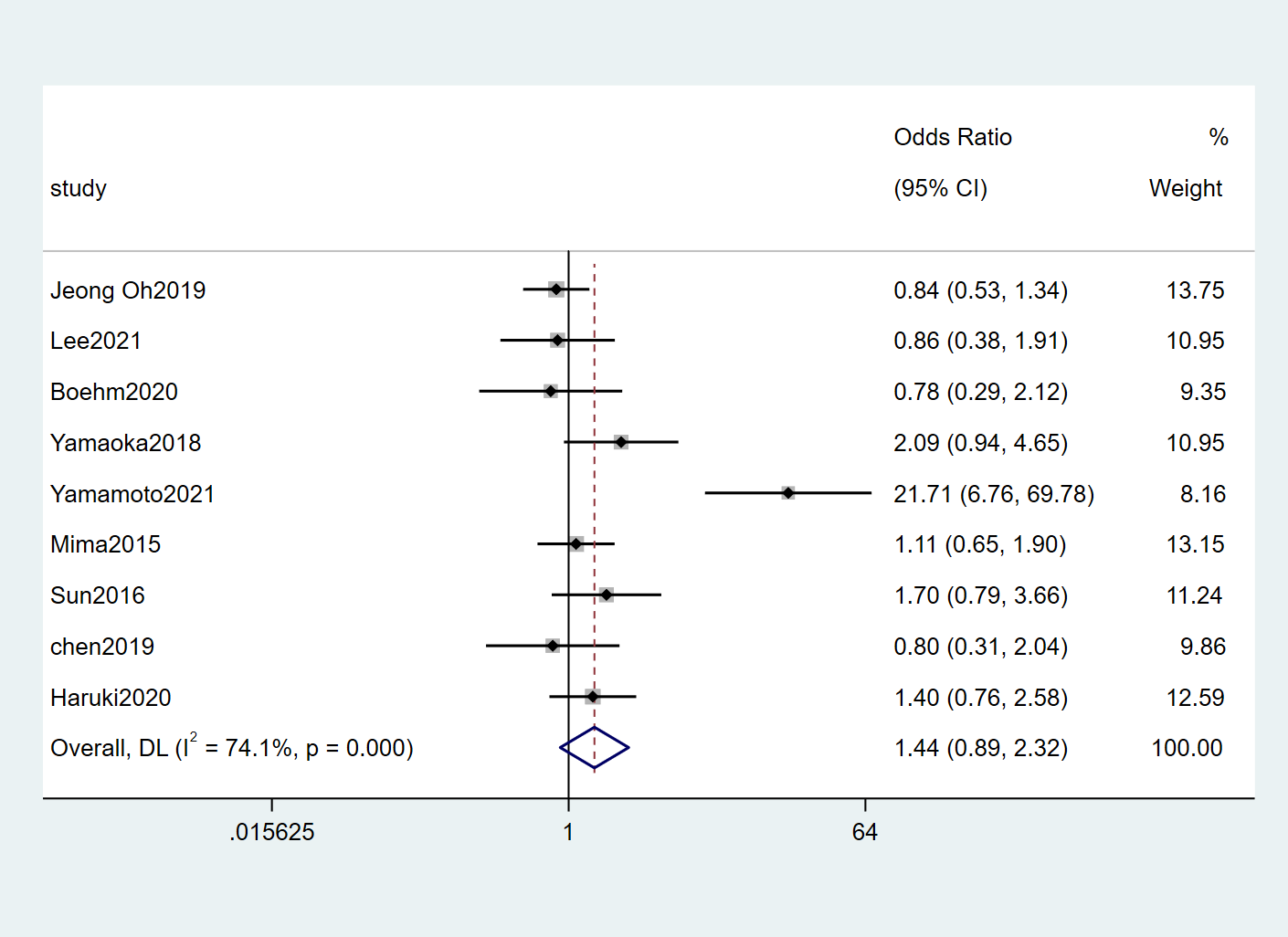

Supplement: Supplementary file 9 [file Image_7.TIF]
